# Development and validation of an administrative data algorithm to identify adults who have endoscopic sinus surgery for chronic rhinosinusitis

**DOI:** 10.1186/s40463-017-0216-0

**Published:** 2017-05-08

**Authors:** Kristian I. Macdonald, Shaun J. Kilty, Carl van Walraven

**Affiliations:** 1 MD FRCSC, Department of Otolaryngology – Head & Neck Surgery, University of Ottawa, Ottawa, ON Canada; 2 MD MSc FRCPC, Department of Medicine, Ottawa Hospital Research Institute, University of Ottawa, Ottawa, ON Canada

**Keywords:** Chronic rhinosinusitis, Administrative database research, Endoscopic sinus surgery, Diagnostic codes

## Abstract

**Background:**

This was a diagnostic accuracy study to develop an algorithm based on administrative database codes that identifies patients with Chronic Rhinosinusitis (CRS) who have endoscopic sinus surgery (ESS).

**Methods:**

From January 1^st^, 2011 to December 31^st^, 2012, a chart review was performed for all hospital-identified ESS surgical encounters. The reference standard was developed as follows: cases were assigned to encounters in which ESS was performed for Otolaryngologist-diagnosed CRS; all other chart review encounters, and all other hospital surgical encounters during the timeframe were controls. Algorithm development was based on International Classification of Diseases, version 10 (ICD-10) diagnostic codes and Canadian Classification of Health Interventions (CCI) procedural codes. Internal model validation was performed with a similar chart review for all model-identified cases and 200 randomly selected controls during the following year.

**Results:**

During the study period, 347 cases and 185,007 controls were identified. The predictive model assigned cases to all encounters that contained at least one CRS ICD-10 diagnostic code and at least one ESS CCI procedural code. Compared to the reference standard, the algorithm was very accurate: sensitivity 96.0% (95%CI 93.2–97.7), specificity 100% (95% CI 99.9–100), and positive predictive value 95.4% (95%CI 92.5–97.3). Internal validation using chart review for the following year revealed similar accuracy: sensitivity 98.9% (95%CI 95.8–99.8), specificity 97.1% (95%CI 93.4–98.8), and positive predictive value 96.9% (95%CI 93.0–99.8).

**Conclusion:**

A simple model based on administrative database codes accurately identified ESS-CRS encounters. This model can be used in population-based cohorts to study longitudinal outcomes for the ESS-CRS population.

## Background

Chronic Rhinosinusitis (CRS) is a common and debilitating inflammatory disease of the sinonasal cavities. CRS is associated with significant resource utilization and burden on health care expenditures [1]. The prevalence of CRS has been quoted as between 5 and 15% of the population [1, 2], and appears to be rising [3]. Patients with CRS self-report their overall health status at a level similar to those with other chronic diseases including current or previous cancer, asthma, migraine, arthritis and epilepsy [4].

Much of the epidemiological data that forms our understanding of CRS is based on studies that identify CRS within large administrative databases and health surveys. We recently published a systematic review of studies that determined the accuracy of these methods to identify CRS [5], and found three studies that compared CRS identification (ascertained from diagnostic codes and self-reporting) to a reference standard (including clinician-performed chart review, nasal endoscopy, and Otolaryngologist-based CRS clinical diagnosis), with moderate to good accuracy.

Health administrative (HA) data may provide the best research modality to develop reliable population-based statistics for CRS patients. HA data have great potential to answer important research questions because of their low cost (since the data are already collected), wide external validity (since the data can cover all people within a particular health care system), and large numbers of patients to provide statistical power [6].

Administrative databases are not built for research purposes, and HA research “*creates risks that can make them uninterpretable or bias their results*” [7]. Within HA data, diseases and procedures are represented with codes. The validity of using HA data to answer research questions is dependent on the accuracy of these codes for the entity they are supposed to represent. Inaccuracies via coding errors that occur in defining the initial cohort, the exposure, or the outcome in an administrative data project can result in biased conclusions. Despite the importance of establishing the accuracy of administrative database codes, such validation is performed in less than 20% of administrative database studies [8]. One of the core (and arguably most important) requirements for using ADs for research involves validation of the codes that serve as proxies of a defined population [9, 10].

Our objective was to identify a model that that would accurately identify CRS patients within HA data. The single physician diagnostic CRS code “473.x” (Version 9 of the International Classification of Disease (ICD-9)) is one such model for identifying CRS cases. However, the aforementioned systematic review identified one study in which this code had just a 34% positive predictive value (PPV). A more feasible solution to meet our objective of capturing a CRS cohort within HA data was to examine CRS patients who had ESS. CRS patients who fail medical therapy are potential surgical candidates, and this subgroup therefore represents patients with medically refractory CRS [11].

We first created a chart review-based reference standard cohort of patients who had ESS for CRS, and then derived a model based on health administrative data to identify this population within a surgical cohort. The final objective was a model that, when applied to all surgical encounters, accurately identified ESS-CRS encounters.

## Methods

This was a validation study of diagnostic test accuracy using several measures of accuracy including sensitivity, specificity and predictive values. To achieve current standards in performing studies of diagnostic accuracy, we adhered to the Standards for Reporting of Diagnostic Accuracy Studies (STARD, 2015 version [12], Appendix). This study received institutional research ethics board approval (OHSN-REB 20140164).

### Databases used

The Ottawa Hospital Data Warehouse (OHDW) contains data from several source systems of patient data dating back as far as 1996 for patients treated at the Ottawa Hospital (TOH), a 1000-bed tertiary care hospital serving over 1.2 million patients and affiliated with the University of Ottawa. Several groups of variables are recorded for each patient encounter including unique identifiers, patient demographics, encounter type, diagnoses, and services rendered (including surgeries). We used the surgery dataset, an online computerized charting and scheduling system for all operations that occur at TOH back to April 2008, having several checklists in place to ensure the correct surgery for the correct indication is recorded (such as the surgeon completing and submitting the paperwork for the surgery, the actual procedure(s) that was (were) performed during the operation), all of which is confirmed by the surgeon at the end of the case.

While the OHDW contains data for TOH patients, the Institute for Clinical Evaluative Sciences (ICES) maintains administrative data for over 13 million people covered by the publically funded health plan. Patients treated at TOH can be identified and linked through both databases with unique identifiers.

### Identifying patients undergoing ESS for CRS at TOH

We obtained a cohort of all TOH surgical encounters that were recorded as Otolaryngologist-performed ESS procedures between January 1^st^, 2011 and December 31^st^, 2012 for patients ≥18 years old. The encounters selected for the chart review were identified as follows: because ESS is only performed by Otolaryngologists, we first identified all surgical encounters performed by this type of surgeon. We then selected all encounters that listed ESS as at least a minor component of the surgery performed during that encounter.

The extracted cohort therefore included all ESS surgeries performed by TOH Otolaryngologists, meaning that all other surgeries conducted at TOH during this time period (all by non Otolaryngologists) were not ESS.

### Chart review: determine whether ESS was conducted for CRS

A chart review was performed of all Otolaryngologist-performed ESS cases to identify those in which ESS was the predominant surgery performed (as opposed to other procedures such as open sinus approaches), and in which ESS was performed for Otolaryngologist-diagnosed CRS (as opposed to other indications such as benign tumours, cerebrospinal fluid leaks, encephaloceles, trauma, foreign bodies, and invasive fungal sinusitis) [13–15]. The chart review was performed by a single author (KM), and involved analysis of primary care physician referrals, clinic notes, operative notes, and sinus CT imaging. We used Otolaryngologist-diagnosed CRS as opposed to a retrospective chart review to identify symptoms and objective findings meeting CRS diagnostic criteria [11], because the latter approach would more likely result in incomplete data collection and misclassification. If the listed diagnosis was recurrent sinusitis, a more detailed chart review was performed to determine if the patient had coexisting CRS. This included clinic notes, preoperative imaging, and prior OR reports. If the patient had associated CRS, the encounter was labeled as a case, otherwise a control.

Patient encounters in which the chart review confirmed ESS for CRS were categorized as cases. All other encounters were categorized as controls.

### Linkage to population-based datasets at ICES

This dataset was linked to ICES via unique identifiers that were encrypted to maintain patient confidentiality. This linked dataset with assigned ESS-CRS cases and controls then provided the reference standard from which the predictive model was created.

### Derivation and internal validation of model to identify ESS for CRS encounters

The same clinician (KM) who performed the chart review created the model. Model development was based on an *a priori* identification of codes that could differentiate cases and controls. Table 1 lists the ICD-10 (International classification of diseases, version 10 [16]) diagnostic codes for CRS and CCI (Canadian Classification of Health Interventions, version 2015 [17]) codes for ESS that were identified from this process.

**Table 1:** Administrative database codes used in predictive model

Chronic Rhinosinusitis			Endoscopic sinus surgery	
Diagnosis	ICD-10 code*	ICD-09 code*	Procedure	CCI code*
Chronic sinusitis	J32	473	Therapeutic Interventions on the Ethmoidal Sinus	1.EU.^^.^^
Chronic maxillary sinusitis	J32.0	473.0	Drainage, ethmoidal sinus	1.EU.52.^^
Chronic frontal sinusitis	J32.1	473.1	Excision partial, ethmoidal sinus	1.EU.87.^^
Chronic ethmoidal sinusitis	J32.2	473.2	Therapeutic Interventions on the Sphenoidal Sinus	1.EV.^^.^^
Chronic sphenoidal sinusitis	J32.3	473.3	Drainage, sphenoidal sinus	1.EV.52.^^
Chronic pansinusitis	J32.4	N/A	Excision partial, sphenoidal sinus	1.EV.87.^^
Other chronic sinusitis	J32.8	473.8	Therapeutic Interventions on the Maxillary Sinus	1.EW.^^.^^
Chronic sinusitis, unspecified	J32.9	473.9	Excision partial, sphenoidal sinus	1.EV.87.^^
Nasal polyp	J33	471	Therapeutic Interventions on the Maxillary Sinus	1.EW.^^.^^
Polyp of nasal cavity	J33.0	471.0	Drainage, maxillary sinus	1.EW.52.^^
Polypoid sinus degeneration	J33.1	471.1	Therapeutic Interventions on the Frontal Sinus	1.EX.^^
Other polyp of sinus	J33.8	471.8	Drainage, frontal sinus	1.EX.52.^^
Nasal polyp, unspecified	J33.9	471.9	Destruction, frontal sinus	1.EX.59.^^
			Repair, frontal sinus	1.EX.80.^^
			Excision partial, frontal sinus	1.EX.87.^^
			Therapeutic Interventions on the Paranasal Sinuses	1.EY.^^.^^
			Excision partial, paranasal sinuses	1.EY.87.^^
			Excision radical, paranasal sinuses	1.EY.91.^^

Codes used in predictive model to identify patients who had endoscopic sinus surgery for chronic rhinosinusitis, with a chart review as a reference standard

International Classification of Diseases, version 10; *CCI* Canadian Classification of Health Interventions

Model variations were developed in a trial and error approach. We considered several variable types for model inclusion, including hospital length of stay (as most ESS is day surgery), age and major comorbidities (because ESS for CRS is usually an elective surgery that may be performed in younger and healthier people compared to other major surgeries), and the CCI and ICD-10 codes listed in Table 1. Our aim was to develop a simple model that used as few codes and variable types as possible, but that made clinical sense. We theorized that each ESS-CRS surgical encounter should contain some variation of ICD-10 CRS and CCI ESS codes, and so we determined to use at least these two variable types in our model. The model was built and adjusted based on comparing the accuracy of model case ascertainment to the reference standard.

The final model accuracy was displayed in a 2x2 table comparing the case status of the model output to the reference standard. Validation statistics with 95% confidence intervals (95% CI) were calculated, using SAS version 9.3 for UNIX (SAS Institute, Inc., USA).

Internal validation was then performed to determine model accuracy within another TOH cohort from a different time-period. Using model criteria, all TOH patient encounters identified by the model as cases and 200 randomly selected controls between Jan. 1^st^, 2013 and Dec. 31^st^, 2013 were retrieved. A chart review was performed of the approximate 400 encounters to determine reference standard case status, by the same clinician, (KM) blinded to the model-predicted case status. Once the chart review was completed, model case status was revealed, and another 2x2 table and set of validation statistics were created to determine internal validation of the model.

## Results

### Chart review

**Fig. 1:**
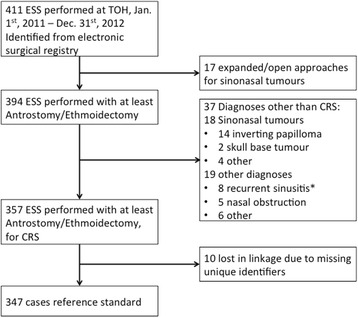
Flow chart of chronic rhinosinusitis - endoscopic sinus surgery chart review. Chart review was performed for TOH surgical encounters in which ESS was performed during the defined time period. ESS = endoscopic sinus surgery; CRS = chronic rhinosinusitis; TOH = The Ottawa Hospital

From Jan. 1^st^ 2011, to Dec. 31^st^, 2012, 411 TOH surgical encounters were identified as having ESS (Fig. 1). Of these, 17 were excluded after the chart review revealed that the major surgery was one other than ESS, leaving 394 encounters that included at least endoscopic antrostomy and ethmoidectomy. Another 37 encounters were excluded because the procedures were for diagnoses other than chronic sinusitis, including 18 sinonasal tumours and 8 with recurrent sinusitis with no evidence of associated CRS. The OR report (with the surgery and indication) for the specified surgical encounter was sufficient to establish case status in all but the 8 patients with recurrent sinusitis. For these 8 patients, no satisfactory evidence of associated CRS could be determined from the chart review. This resulted in 357 ESS-CRS cases during the study period.

### Linkage of chart review data to ICES dataset

Patient encounters within the TOH chart review cohort and ICES dataset were linked via encrypted unique identifiers. Thirteen patients (ten cases and three controls) were lost in the linkage due to missing unique identifiers. The linked dataset contained 185,354 hospital encounters representing all surgeries performed at TOH from Jan. 1^st^, 2011, to Dec. 31^st^, 2012. This linked dataset, with 347 cases and 185,007 controls, (case prevalence = 0.19) was used to develop the predictive model.

### Model development

The model was created through a trial and error approach, using variables within the linked dataset. It was evident from analyzing the variable types and values that each case encounter contained commonly assigned CRS diagnostic and ESS procedural codes. The first model assigned cases if an encounter listed any of the ICD-10 CRS diagnostic codes listed in Table 1. Compared to the reference standard case ascertainment, this model had excellent validation statistics: sensitivity 96.5% (95% CI 93.9–98.1) and positive predictive value (PPV) 93.3% (95% CI 90.1–95.6).

The second model was based on procedural ESS codes only. Cases were assigned if an encounter listed any one of the CCI ESS procedural codes listed in Table 1. Compared to the reference standard case ascertainment, this model had similarly high validation statistics: sensitivity 96.8% (95%CI 94.2–98.3) and PPV 93.3% (95%CI 90.1–95.6).

The third and final model combined features from the first two models, resulting in a slightly improved PPV. Encounters were classified by the final model as ESS for CRS if they had been coded with any of the ICD-10 CRS diagnostic codes listed in Table 1 along with any of the CCI ESS surgical codes listed in Table 1. All encounters not meeting these criteria were classified as controls (i.e. not ESS for CRS). Table 2 compares validation statistics of the three model variations. Specificity for all three models was 100%.

**Table 2:** Comparison of validation statistics of three models to predict CRS-ESS encounters

Model version	Sensitivity % (95%CI)	Specificity % (95%CI)	PPV % (95%CI)
#1: Any CRS diagnostic ICD-10 code	96.5 (93.9–98.1)	100 (99.9–100)	93.3 (90.1–95.6)
#2: Any ESS procedural CCI code	96.8 (94.2–98.3)	100 (99.9–100)	93.3 (90.1–95.6)
#3: #1 AND #2	96.0 (93.2–97.7)	100 (99.9–100)	95.4 (92.5–97.2)

Comparison of three different model versions to predict CRS-ESS status, compared to the reference standard

*CI* confidence interval, *CRS* Chronic rhinosinusitis, *ESS* Endoscopic sinus surgery, *PPV* positive predictive value

**Fig. 2:**
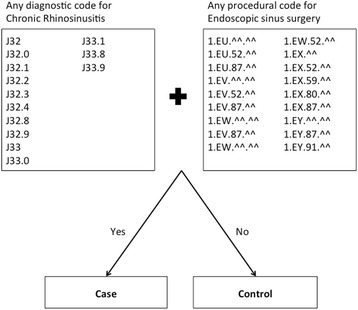
Overview of final model to identify CRS-ESS case encounters within a surgical cohort. Case was assigned if an encounter contained one of the ICD-10 diagnostic CRS codes, AND one of the CCI procedural ESS codes. CRS = Chronic Rhinosinusitis; ESS = Endoscopic sinus surgery; ICD-10 = International Classification of Diseases, version 10; CCI = Canadian Classification of Health Interventions

Table 3 displays a 2x2 table comparing the final model output to the reference standard, with validation statistics including sensitivity 96.0% (95%CI 93.2–97.7), specificity 100% (95%CI 99.9–100), positive predictive value 95.4% (95%CI 92.5–97.3), positive likelihood ratio 11,096 (95%CI 6,794–18,120), and negative likelihood ratio 0.04 (95%CI 0.02–0.07). Fig. 2 displays a graphical overview of the final model.

**Table 3:** Predictive model vs reference standard for ESS-CRS status

Model output	Reference standard		Total
	Case	Control	
Case	333	16	349
Control	14	184,991	185,005
Total	347	185,007	185,354

Predictive model based on administrative database codes

*CCI* Canadian Classification of Health Interventions, *CRS* Chronic rhinosinusitis, *ESS* Endoscopic sinus surgery, *ICD-10* International Classification of Disease, version 10

Further examination of the 16 false positives (encounters identified as cases by the model but were controls by the reference standard), revealed that eight were patients with recurrent sinusitis according to the reference standard.

### Internal validation of model

Using criteria from the final model, we retrieved a hospital cohort of all cases and 200 randomly selected controls from year following the derivation cohort, Jan. 1^st^, 2013 to Dec. 31^st^, 2013. A chart review, blinded to model output case status, was then performed to determine reference standard case status. The OR report for the selected surgical encounter was sufficient to determine case status in all encounters. After the model output case status was revealed, a 2x2 table was again created with excellent accuracy: sensitivity 98.9% (95%CI 95.8–99.8) specificity 97.1% (95%CI 93.4–98.8), positive predictive value 96.9% (95%CI 93.0–98.7) positive likelihood ratio 33.6 (95%CI 15.3–74.0), and negative likelihood ratio of 0.01 (95%CI 0.00–0.04). (Table 4)

**Table 4:** Internal validation of predictive model of ESS-CRS status

Model output	Reference standard		Total
	Case	Control	
Case	186	6	192
Control	2	198	200
Total	188	204	392

The ESS-CRS predictive model was applied to all TOH surgical patients in 2013. Sensitivity 98.9% specificity 97.1%, positive predictive value 96.9%

*CRS* Chronic rhinosinusitis, *ESS* Endoscopic sinus surgery, *TOH* The Ottawa Hospital

## Discussion

We developed an internally validated model that accurately identified patient encounters in which endoscopic sinus surgery was performed for chronic rhinosinusitis at the Ottawa Hospital over a 3-year period. This model is simple and includes readily available administrative data to accurately differentiate between ESS-CRS cases and controls within a surgical cohort. The criteria for a case (at least one ICD-10 CRS diagnostic code and at least one CCI ESS procedural code) were not created *a priori*, but instead through a trial and error process with the observations and variables contained within the dataset, with knowledge of the chart review data. However, we argue that this model has face validity for Otolaryngologic epidemiology research.

Despite the importance of validation studies for AD codes, the lack of code validation is hardly unique to CRS: a 2011 review of a random Medline sample of 115 AD research studies found that only 14 (12.1%) “measured or referenced the association of the code with the entity is supposedly represented”, and *“of five studies reporting code sensitivity and specificity, the estimated probability of code-related condition in code-positive patients was less than 50% in two”* [8]. Therefore, *“people with a code frequently do not have the condition it represents”.* Applying this to our population, it is incorrect to assume ESS and CRS codes are accurate without measuring the ability of a code to differentiate between a case and a control, with an acceptable reference standard.

Validation studies like this one are essential for future AD research using specific codes. As one example, a recent publication used a similar chart review method used to validate National Surgical Quality Improvement Program 30-day readmission codes [18].

Conducting such health administrative database research in Ontario is aided by the fact that ESS is a publically funded procedure. As a result, all ESS performed in Ontario should be captured within these databases. Advantages of this population-based method to identify patients undergoing ESS for CRS include: 1) minimal cost, as most work for this research is at the computer and through a chart review; 2) large numbers of patients from a population-based database allow complete analyses without sampling; and 3) if externally validated, this model can be used to study longitudinal outcomes for ESS as an intervention in CRS patients.

Others have identified ESS procedures (for all indications, not just CRS) in HA databases using similar procedural codes for ESS (similar CCI codes in Alberta [19], and Common Procedural Terminology codes in the US [20]). In these studies, the authors did not attempt to determine code accuracy to determine if patients identified by these methods actually had ESS. Our chart review revealed that 17/411 (4%) patients who were identified as having ESS actually had a more invasive open procedure, and 37/394 (9.4%) patients who had at least endoscopic antrostomy/ethmoidectomy did not have CRS. Combined, 54/411 (13.1%) patients who were coded at TOH as having ESS did not truly have ESS or CRS. However, despite these potential inadequacies in code accuracy, a model based only on ESS codes achieved almost the same accuracy in identifying ESS-CRS cases as our final model (sensitivity 96.8% (95%CI 94.2–98.3), specificity 100% (95%CI 99.9–100), PPV 93.3% (95%CI 90.1–95.6)), giving validity to previous authors’ work. In an analysis similar to ours (although again without an attempt at code validation), Benninnger et al. identified ESS-CRS patients within a cohort of 35.5 million patients enrolled in the Market Scan Commercial Claims and Encounter database in 2010 [21]. They used analogous codes: sinus surgery codes (CPT-4 31254-31288 [Common Procedure Terminology, 4^th^ Ed]), and ICD-9 CRS diagnostic codes (473.X), and identified 2,833 ESS-CRS patients. Our results provide argument that these methods of identifying ESS procedures may be accurate – this statement would be further supported if our model was externally validated or if other authors carried out similar validation projects.

We found that eight of the false positives identified by the final model were encounters in which ESS was performed for recurrent sinusitis with no coexisting CRS. This misclassification reflects a potential inability of administrative database codes to differentiate between these two conditions. Although this did not greatly affect our validation statistics, it could affect external validity, for example in centres where a greater proportion of ESS is performed for recurrent sinusitis.

Several assumptions must be made that could be interpreted as study weaknesses. First, development of the reference standard, predictive model, and internal validation were all performed by the one clinician. This bias could influence case ascertainment in the reference standard and internal validation, as well as variable selection for the final model, falsely elevating the model accuracy. Second, we used Otolaryngologist-diagnosed CRS for reference standard case ascertainment. This infers that the Otolaryngologist correctly diagnosed CRS. It is possible that strict diagnostic criteria were not applied. We considered establishing a guideline-based CRS diagnosis as the reference standard through a retrospective chart review of the patient charts (including clinic notes and imaging) but this would have been exposed to recall and selection bias. Third, we must also assume that patient encounters are correctly recorded in the surgical database, and specifically that ESS encounters were correctly identified for the chart review.

### Future direction

Our future direction includes external validation at other tertiary care centres, similar to the methods used in internal validation. An externally validated model can then be used to study longitudinal outcomes and health services research of this population. Other centres may be encouraged to perform their own external validation based on our model criteria, with the overarching objective of producing much needed accurate CRS epidemiological data.

## Conclusions

A simple model based on administrative database codes accurately identified surgical encounters in which endoscopic sinus surgery was performed for chronic rhinosinusitis (CRS) at a tertiary care centre. Compared to a reference standard including a chart review and Otolaryngologist-diagnosed CRS, this model achieved excellent validation statistics: sensitivity 96.0% (95%CI 93.2–97.7), specificity 100%, and positive predictive value 95.4% (95%CI 92.5–97.3). Internal validation was achieved with similarly high validation statistics.

This model has potential for large population-based cohorts to study longitudinal outcomes of patients who have endoscopic sinus surgery for chronic rhinosinusitis.

## Appendix

**Table 5:** Standards for reporting diagnostic accuracy studies checklist [12] (2015 version)

Section & Topic	No	Item	On page & line
Title or Abstract	1	Identification as a study of diagnostic accuracy using at least one measure of accuracy (such as sensitivity, specificity, predictive values, or AUC)	P6 L2
Abstract	2	Structured summary of study design, methods, results, and conclusions	P2
Introduction	3	Scientific and clinical background, including the intended use and clinical role of the index test	P4
	4	Study objectives and hypotheses	P5 L9
Methods			
*Study design*	5	Whether data collection was planned before the index test and reference standard were performed (prospective study) or after (retrospective study)	P7 L5
*Participants*	6	Eligibility criteria	P7 L5
	7	On what basis potentially eligible participants were identified (such as symptoms, results from previous tests, inclusion in registry)	P7 L5
	8	Where and when potentially eligible participants were identified (setting, location and dates)	P7 L5
	9	Whether participants formed a consecutive, random or convenience series	P7 L5
*Test methods* *Analysis*	10a	Index test, in sufficient detail to allow replication	P8 L13
	10b	Reference standard, in sufficient detail to allow replication	P7 L10
	11	Rationale for choosing the reference standard (if alternatives exist)	P8 L13
	12a	Definition of and rationale for test positivity cut-offs or result categories of the index test, distinguishing pre-specified from exploratory	P8 19
	12b	Definition of and rationale for test positivity cut-offs or result categories of the reference standard, distinguishing pre-specified from exploratory	P8 L19
	13a	Whether clinical information and reference standard results were available to the performers/readers of the index test	P8 L19
	13b	Whether clinical information and index test results were available to the assessors of the reference standard	P8 L20
	14	Methods for estimating or comparing measures of diagnostic accuracy	P9 L3
	15	How indeterminate index test or reference standard results were handled	N/A
	16	How missing data on the index test and reference standard were handled	N/A
	17	Any analyses of variability in diagnostic accuracy, distinguishing pre-specified from exploratory	N/A
	18	Intended sample size and how it was determined	N/A
Results			
*Participants*	19	Flow of participants, using a diagram	Fig. 1
	20	Baseline demographic and clinical characteristics of participants	N/A
	21a	Distribution of severity of disease in those with the target condition	N/A
	21b	Distribution of alternative diagnoses in those without the target condition	P11 L21
	22	Time interval and any clinical interventions between index test and reference standard	N/A
*Test results*	23	Cross tabulation of the index test results (or their distribution) by the results of the reference standard	Tables 3 & 4
	24	Estimates of diagnostic accuracy and their precision (such as 95% confidence intervals)	Tables 3 & 4
	25	Any adverse events from performing the index test or the reference standard	
Discussion			
	26	Study limitations, including sources of potential bias, statistical uncertainty, and generalisability	P15 L19
	27	Implications for practice, including the intended use and clinical role of the index test	P17 L3
Other Information	28	Registration number and name of registry	N/A
	29	Where the full study protocol can be accessed	P17 L8
	30	Sources of funding and other support; role of funders	P17 L8
